# The Endothelin Receptor Antagonist Macitentan Inhibits Human Cytomegalovirus Infection

**DOI:** 10.3390/cells10113072

**Published:** 2021-11-08

**Authors:** Natalia Landázuri, Jennifer Gorwood, Ylva Terelius, Fredrik Öberg, Koon Chu Yaiw, Afsar Rahbar, Cecilia Söderberg-Nauclér

**Affiliations:** 1 Microbial Pathogenesis Unit, Department of Medicine Solna, Karolinska Institutet, SE-171 76 Stockholm, Sweden; natalia.landazuri@disstockholm.se (N.L.); jennifer.gorwood@ki.se (J.G.); koon.chu.yaiw@ki.se (K.C.Y.); afsar.rahbar@ki.se (A.R.); 2 Division of Neurology, Karolinska University Hospital, SE-171 76 Stockholm, Sweden; 3 Medivir AB, SE-141 22 Huddinge, Sweden; ylva.terelius@gmail.com (Y.T.); Fredrik.Oberg@medivir.com (F.Ö.)

**Keywords:** cytomegalovirus, endothelin receptor, repurposing

## Abstract

Human cytomegalovirus (HCMV) infection is an important cause of morbidity and mortality in immunocompromised patients and a major etiological factor for congenital birth defects in newborns. Ganciclovir and its pro-drug valganciclovir are the preferred drugs in use today for prophylaxis and treatment of viremic patients. Due to long treatment times, patients are at risk for developing viral resistance to ganciclovir and to other drugs with a similar mechanism of action. We earlier found that the endothelin receptor B (ETBR) is upregulated during HCMV infection and that it plays an important role in the life cycle of this virus. Here, we tested the hypothesis that ETBR blockade could be used in the treatment of HCMV infection. As HCMV infection is specific to humans, we tested our hypothesis in human cell types that are relevant for HCMV pathogenesis; i.e., endothelial cells, epithelial cells and fibroblasts. We infected these cells with HCMV and treated them with the ETBR specific antagonist BQ788 or ETR antagonists that are approved by the FDA for treatment of pulmonary hypertension; macitentan, its metabolite ACT-132577, bosentan and ambrisentan, and as an anti-viral control, we used ganciclovir or letermovir. At concentrations expected to be relevant in vivo, macitentan, ACT-132577 and BQ788 effectively inhibited productive infection of HCMV. Of importance, macitentan also inhibited productive infection of a ganciclovir-resistant HCMV isolate. Our results suggest that binding or signaling through ETBR is crucial for viral replication, and that selected ETBR blockers inhibit HCMV infection.

## 1. Introduction

Human cytomegalovirus (HCMV) is a ubiquitous, opportunistic double-stranded DNA virus of the herpesviridae family [1]. Depending on geographical location and socioeconomic status, 40% to >90% of the population is infected with HCMV [1]. After a primary HCMV infection, which is typically asymptomatic, the virus establishes life-long latency and persistence. Latent HCMV has no obvious complications in otherwise healthy people. However, in immunocompromised individuals, such as AIDS patients and transplant patients, reactivation of HCMV can lead to significant morbidity and mortality [1]. HCMV establishes latency in myeloid lineage progenitor cells and can be transferred from donors to recipients of solid organ and bone marrow transplants [2,3]. The virus can be reactivated when monocytes differentiate into macrophages or dendritic cells by inflammatory stimuli [4,5,6], which may occur as a consequence of organ or stem cell graft rejection. Transplant recipients can hence acquire a primary infection from an HCMV-positive donor [2,3,7]. 

During acute infection, epithelial cells are major portal of entry and a main source of HCMV dissemination. HCMV is commonly excreted from epithelial cells and spreads to other individuals through saliva, urine, breast milk, and genital excretions [8]. Endothelial cells lining the vascular tree are also a primary source of HCMV entry and mediate viral spread to different organs during acute infection. Fibroblasts are easily infected with HCMV [9] and also represent target cells in widespread HCMV disease. In AIDS patients, HCMV infection of retinal epithelial cells is a major HCMV-driven complication causing retinitis, which can lead to blindness [10,11]. Moreover, HCMV infection is the most common etiological agent for congenital birth defects in newborns and is implicated in cardiovascular disease and cancer [12,13,14]. 

Patients at risk for HCMV-related complications are treated with antivirals either prophylactically for at least 3 months or as a pre-emptive therapy when frequent PCR monitoring shows evidence of HCMV replication in the patient [3]. Ganciclovir and its per oral pro-drug valganciclovir are long-standing first-line systemic drugs in use for prophylaxis and treatment of HCMV infections [15]. Ganciclovir, a deoxyguanosine analog, is phosphorylated once by the viral kinase encoded by UL97 and then twice by cellular kinases. The active form, ganciclovir triphosphate, is incorporated into the elongating DNA and inhibits the viral DNA polymerase encoded by the HCMV gene UL54 [15,16]. Mutations arising in the UL97 or UL54 genes often mediate resistance to ganciclovir [17,18].

Ganciclovir resistance may develop after treatment for more than 3–4 months, and necessitate the use of alternative drugs [3]. Extended treatment can also lead to myelosuppression [19], a detrimental side effect for hematopoietic stem cell transplant patients or for patients treated with myelosuppressive drugs to prevent graft-versus-host disease. Ganciclovir is therefore not suitable for prophylactic use in this group of patients. Second-line drugs for systemic therapy also target the UL54 viral DNA polymerase. These include cidofovir, a nucleotide analog of cytidine, which only requires phosphorylation by cellular enzymes, and Foscavir, a pyrophosphate analog that prevents incorporation of dNTPs into the viral DNA polymerase [3]. Ganciclovir, cidofovir and Foscavir have similar mechanisms of action and cross-resistance has been reported [16,20]. Letermovir, which targets the viral terminase complex [21], was approved for HCMV prophylaxis and treatment in 2017 [22]. This was the first drug to be approved for HCMV treatment since 2003. Therefore, additional clinically approved drugs are needed that target other critical steps of HCMV infection, which can be used especially in patients infected with drug-resistant strains.

Previously, we found that HCMV infection upregulates the endothelin receptor type B (ETBR) at the transcriptional and protein levels in endothelial and smooth muscle cells [23], which raised the possibility that this receptor or the endothelin axis may play an important role during HCMV infection. In support of this hypothesis, we recently found that HCMV infection inhibits ET-1 transcript expression, and via downregulation of endothelin converting enzyme-1 (ECE-1) that cleaves the ET-1 precursor protein to mature ET-1, it also inhibits release of the ET-1 peptide [24]. ET-1 is the most common isoform peptide of endothelin and acts as a very potent vasoconstrictor. ET-1 provides its effect through binding to two G-protein-coupled receptor subtypes: ETAR and ETBR [25], which have opposite functions on vascular tone. Binding to both receptors on vascular smooth muscle cells leads to vasoconstriction, while binding to ETBR on endothelial cells leads to clearance of ET-1 and release of nitric oxide and prostacyclin, and consequent vasodilatation [25]. ETBR thus helps to clear endothelin-1 (ET-1) from the circulation.

ET-1 concentration is often elevated in patients with cardiovascular diseases, which can lead to vasoconstriction and hypertension [26]. Endothelin receptor (ETR) antagonists have been developed to treat pulmonary hypertension [27]. Three different ETR antagonists are today FDA-approved for the treatment of pulmonary hypertension: bosentan, macitentan, and ambrisentan [28,29,30,31]. Bosentan and macitentan target both ETAR and ETBR, while ambrisentan targets ETAR.

Here, we tested the hypothesis that an ETR antagonist could be repurposed to treat HCMV infections by testing their effects to prevent HCMV infection in clinically relevant cell types: endothelial cells, epithelial cells, and fibroblasts. We found that ETBR antagonists can inhibit HCMV infection in various cell types.

## 2. Materials and Methods

### 2.1. Materials

BQ788, ACT-132577, macitentan, and bosentan were from MedChemExpress LCC (MedChemExpress LCC, Princeton, NJ, USA). Ambrisentan was from Ark Pharm Inc. (Ark Pharm Inc., Microsoft Libertyville, IL, USA). Ganciclovir was from Hoffmann La Roche (Stockholm, Sweden). Human primary RPE-1 retinal pigment epithelial cells (a kind gift from Dr. Richard J. Stanton and Dr. Derrick Dargan, Medical Research Council Centre for Virology, UK [32,33]) were cultured in RPMI with 10% fetal bovine serum, penicillin (100 U/mL), and streptomycin (100 µg/mL). Human MRC5 fibroblasts (ATCC, US) were cultured in minimum essential medium with 10% fetal bovine serum, penicillin (100 U/mL), and streptomycin (100 µg/mL). Primary human umbilical vein endothelial cells (HUVECs) were harvested with collagenase as described [34] or purchased (Lonza, Basel, Switzerland) and cultured in endothelial cell growth medium (Lonza). All cells were cultured at 37 °C in 5% CO_2_/95% air.

### 2.2. Cell Viability and Toxicity

Cells were plated on 96-well plates and treated the next day with BQ788, ACT-132577, macitentan, bosentan, ambrisentan, or ganciclovir (0–25 μM). Cell proliferation was evaluated with the CellTiter 96 AQueous Non-Radioactive Cell Proliferation Assay (Promega Biotech, Nacka, Sweden) according to the manufacturer’s instructions.

### 2.3. Viral Infectivity

Cells were plated on chamber slides. The next day, cell monolayers were treated with the aforementioned chemical compounds and infected with HCMV strain VR1814 (titer of 2.5 × 10^6^ pfu/mL) at multiplicity of infection (MOI) of 0.1 (or MOI of 2 when it’s indicated in the text). The ganciclovir-resistant clinical isolate C17222 [35] was propagated in MRC5 fibroblasts. As this isolate is highly cell-associated, we infected HUVECs by exposing them to C17222 infected MRC5s at a HUVEC: infected MRC5 ratio of 100:1; 50:1 or 1:1, respectively. Infected cells were treated or not with 10 µM of ganciclovir, 10 µM of letermovir, 100 µM of BQ788 or 25 µM of macitentan, respectively. As a control, we verified that the infected MRC5 cells could no longer proliferate. Cells were immunostained with an antibody against HCMV immediate-early antigen (targeting exon 2 recognizing IE72, IE86 and IE55 [36], Argene, Biomerieux, Marcy l’Etoile, France) or an antibody against HCMV immediate-early antigen clone 6F8.2 (Merck, Darmstadt, Germany). Antibody binding was visualized with the anti-mouse ImmPRESS kit (Vector Laboratories, Peterborough, UK) or an anti-mouse antibody conjugated with AlexaFluor 488 (Invitrogen, Camarillo, CA, USA). The percentage of IE-positive cells was determined in at least three different images per well in duplicates of each experment. The *t*-test (two-tailed, unpaired) was conducted with Microsoft Excel 2011 (Microsoft, Redmond, WA, USA) or GraphPad Prism (versions 6 or 8, GraphPad Software, San Diego, CA, USA) and used for comparison between a treatment group and the control group, respectively. Differences were considered significant at *p*-values < 0.05.

### 2.4. Viral Output Assay

Cells were plated on 12- or 24-well plates. The next day, we treated the cells with the chemical compounds and infected them with HCMV VR1814 (at multiplicities of infection between 0.1 and 0.2). Three to four days later, the cells were washed with PBS, and the culture medium was replaced with drug-containing fresh medium. Seven days later, the supernatant was harvested. Since the virus produced by RPEs is strongly cell-associated, the cells were scraped and collected with the supernatant. The mixture was sonicated or freeze thawed, and cell debris was pelleted to harvest the supernatant that could contain infectious virus. As cells were incubated with the compounds for several days before collection of the virus-containing supernatant, the presence of active compounds was assumed to be minimal, if any. To account for any remaining active compounds and minimize their action, we diluted the collected supernatants 10-fold in fresh cell culture medium. MRC5 cells, plated on chamber slides, were exposed to the diluted supernatants for 1 or 2 days, fixed, and stained for HCMV-IE as described above. The percentage of IE-positive cells was determined in at least three images for each condition by manual counting. The EC50 for each compound-cell combination was determined in two independent experiments, using GraphPad Prism (versions 6 or 8, GraphPad Software, San Diego, CA, USA) and a non-linear fit of log[inhibitor] vs. response equation with three parameters, a standard slope (Hill Slope = −1.0) and a constraint of the bottom value greater than 0.0. The virus titer was measured by a standard TCID50 method [37] in MRC5 cells with minor modifications. Briefly, the cells were exposed for 5 days to a ten-fold dilution of virus inoculum from either the supernatants or cell-associated virions of HUVEC-infected VR1814 with or without treatment with 100 µM of BQ788 at the indicated time points (i.e. 5 hours after infection and at 3-, 5- and 7-dpi). The cytopathic effects were visualized by IE immunofluorescence staining and the TCID50/mL was calculated as previously described [37].

### 2.5. Plaque Formation Assay

Virus-containing supernatants collected from cells infected in the presence of chemical compounds (as described in the previous paragraph) were serially diluted and used to infect MRC5 cells. Two hours later, the cell culture medium was replaced with cell culture medium containing 0.5% methylcellulose. When plaques formed (10–14 days for HCMV), cells were fixed and stained with 70% methanol and 0.1% methylene blue. Plaques from wells where plaques were clearly distinguishable were counted and the titer of each supernatant was calculated.

### 2.6. Statistical Analysis

All analyses were performed using GraphPad Prism versions 6 or 8 (GraphPad Software, Inc., La Jolla, CA, USA). One-way ANOVA test followed by Dunnett’s multiple comparisons test was used to assess the statistical significance between different variables. Data are presented as the mean ± standard error of the mean. *p* < 0.05 was considered as a statistically significant difference. ****; *p* < 0.0001 ***; *p* < 0.001; ** *p* < 0.01, *; *p* < 0.05. All experiments were performed with three independent repeats.

## 3. Results

### 3.1. ETR Antagonists Are Well Tolerated at Low Concentrations

**Figure 1:**
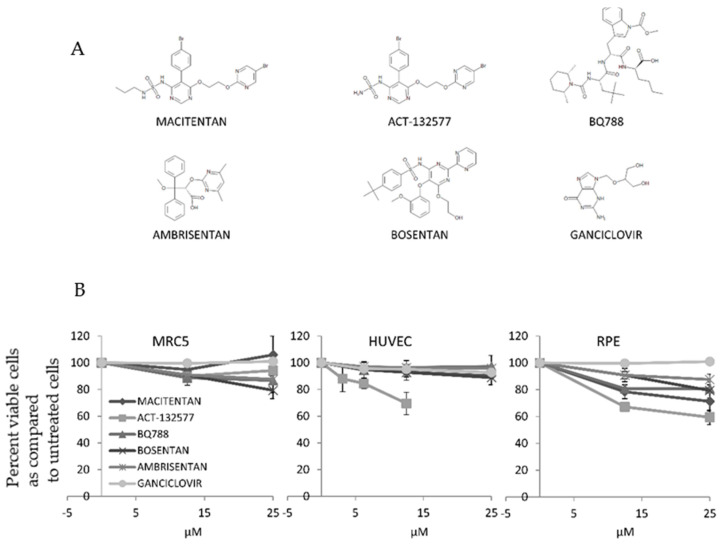
ETR antagonists are not toxic at low concentrations. HUVECs, MRC5, and RPE-1 cells were exposed to various concentrations of macitentan, ACT-132577, BQ788, ambrisentan, bosentan, or ganciclovir. (**A**) Chemical structures of the compounds. (**B**) Cells were treated with the compounds for 7 days, whereafter cell viability was quantified and normalized to untreated cells. ETR antagonists were not toxic for HUVECs, MRC5, and RPE-1 cells at low concentrations. Values are mean ± SD of triplicates.

We first assessed if macitentan, its metabolite ACT-132577, bosentan, ambrisentan, the ETBR-specific chemical compound BQ788, and ganciclovir affected cellular viability in HUVECs (human umbilical cord endothelial cells), MRC5 (human fibroblasts) and RPE-1 cells (human retinal pigment epithelial cells) (Figure 1A). After 7 days of treatment, the compounds did not affect cell viability at the expected peak serum concentration in patients reported in literature [38,39], considering that macintentan has a plasma protein binding capacity exceeding 99%. The most dramatic effect we observed was a reduced cell viability of approximately 30–35% in HUVECs and RPEs treated with the highest concentration of ACT-132577 (Figure 1B and Table 1).

### 3.2. ETR Antagonists Prevent Production of Infectious HCMV

**Figure 2:**
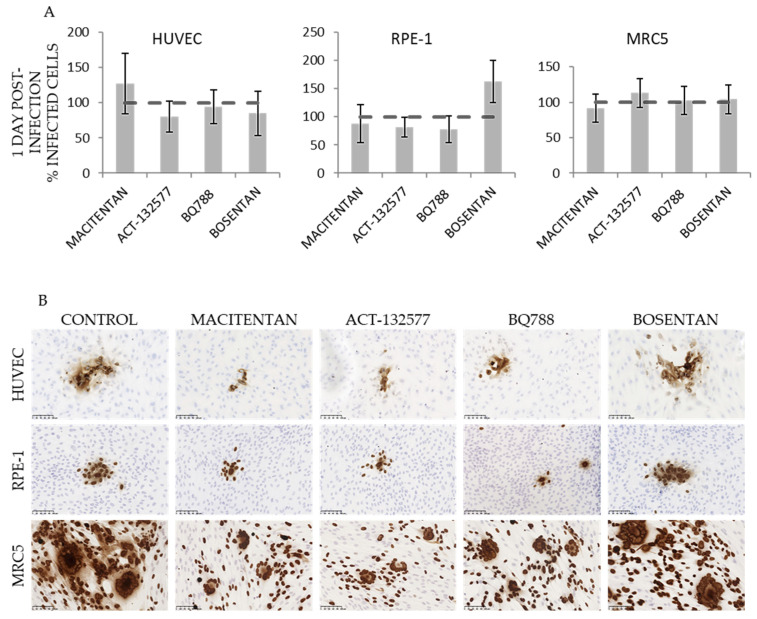
ETR antagonists prevent virion production. HUVECs, RPE-1 and MRC5 cells were treated with macitentan (12.5 μM for HUVEC, 25 μM for RPE and MRC5), ACT-132577 (6.25 μM for HUVEC, 12.5 μM for RPE and MRC5), BQ788 (25 μM), or bosentan (25 μM) and simultaneously exposed to HCMV. Infectivity was assessed by immunostaining for IE. (**A**) Number of IE-positive cells on day 1 post-infection normalized to infected, non-treated cells. (**B**) Representative images of HCMV-driven foci on day 5 after infection. Broken line corresponds to the level of IE expression in infected cells not treated with drugs. Scale bar 100 μM.

To assess the antiviral properties of the different ETR antagonists, we infected HUVECs, MRC5 and RPE-1 cells with HCMV (VR1814 strain) in the presence of macitentan (12.5 μM for HUVEC and 25 μM for RPE-1 and MRC5), ACT-132577 (6.25 μM for HUVEC and 12.5 μM for RPE-1 and MRC5), BQ788 (25 μM), or bosentan (25 μM) and quantified infected cells by their expression of the HCMV IE protein. We did not observe a reduction in the number of IE-positive cells on day 1 after infection (Figure 2A). On day 5, the cell-to-cell spread of the virus appeared to be inhibited by some of the compounds as judged from a reduced formation of IE-positive foci. The IE foci formed in the presence of macitentan, ACT-132577 and BQ788 were visibly smaller than those formed in control or bosentan-treated cells (Figure 2B). This implied that ETR antagonists prevented viral replication or spread.

### 3.3. ETR Antagonists Inhibit HCMV Replictaion by Post Entry Mechanisms

**Figure 3:**
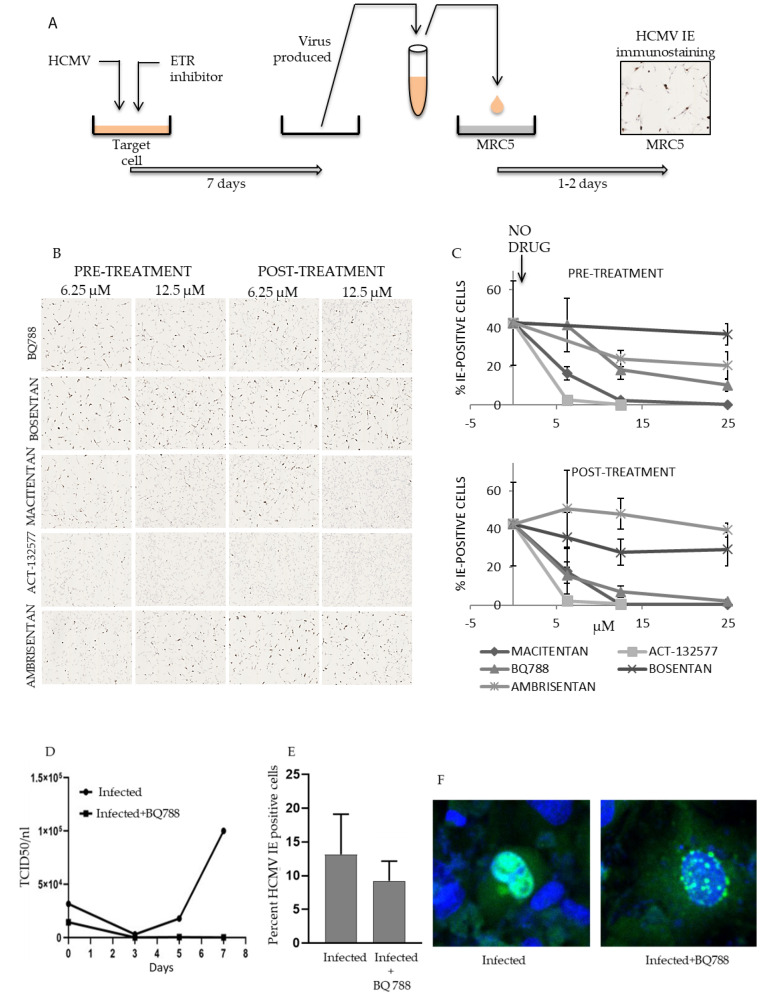
ETR antagonists prevent virus production. HUVECs were treated with ETR antagonists for 1 h before (pretreatment) or after (post-treatment) exposure to HCMV. (**A**–**C**) At seven days post infection, the virus-containing supernatant was harvested and used to infect MRC5 cells. (**A**) Schematic diagram of the experimental procedure. (**B**) Representative images of infected MRC5 fibroblasts 1 day after infection. The brown stain corresponds to IE immunoreactivity. (**C**) Percentage of IE-positive MRC5 cells after 1 day post infection after exposure to supernatant from 7-day infected HUVECs. The arrow indicates data points with no drug (0 μM). (**D**) TCID50/mL was measured in MRC-5 cells. Cells were exposed for 5 days to either supernatants or HUVECs infected with VR1814 (MOI of 2) and treated or not with 100 µM of BQ788 and were collected at 5 h after infection (0-dpi), 3-, 5- and 7-dpi. (**E**) Percentage of positive IE stained cells in HUVECs exposed to C17222 infected MRC-5 cells with ratio 1:50 (*p* = 0.058). (**F**) Representative images of infected and infected and treated with BQ788 at 100 µM with confocal microscopy (×20 magnification). The nuclei are stained with DAPI (blue) and an IE specific antibody (green). Values are mean ± SD of at least triplicate images.

To determine whether blockade of ETR prior to virus entry was necessary to inhibit viral replication, we pretreated HUVECs with ETR antagonists for 1 h before infection with HCMV. As a control, we exposed cells to the virus for 1 h to allow binding and entry of viral particles [40,41] before we added the antagonists to the culture medium. Seven days later, virus-containing supernatants were harvested and used to infect MRC5 fibroblasts (Figure 3A). At one day post infection and before new virions could be produced [42], the percentage of IE-positive MRC5 cells was similar regardless of whether cells had been treated with ETR blockers before or after infection, indicating that the ETR blockers inhibited, to a great extent, post-entry steps of HCMV infection (Figure 3B,C).

At concentrations as low as 6–12 μM of macitentan and its metabolite ACT-132577, dual ETAR and ETBR antagonists, we did not detect any measurable levels of viral production (Figure 3C and Table 2). BQ788, an ETBR-specific antagonist, inhibited viral production by 80% at 12 μM (Figure 3C) and completely at 100 μM (Figure 3D). Ambrisentan, and ETAR-specific antagonist, did not affect viral output (Figure 3C and Table 2), which suggests that the ETAR is not essential for HCMV replication. However, the dual ETAR-ETBR antagonist bosentan also failed to inhibit viral replication (Table 2). The ETBR binding site for bosentan may hence differ from that of macitentan and BQ788. Bosentan either does not interfere with the binding of HCMV proteins to this receptor, or the drug does not affect post entry effects of HCMV involving ETBR signaling.

### 3.4. HCMV Infection Is Inhibited at Low Concentrations of ETR Antagonists in HUVECs, MRC5 and RPE-1 Cells

**Figure 4:**
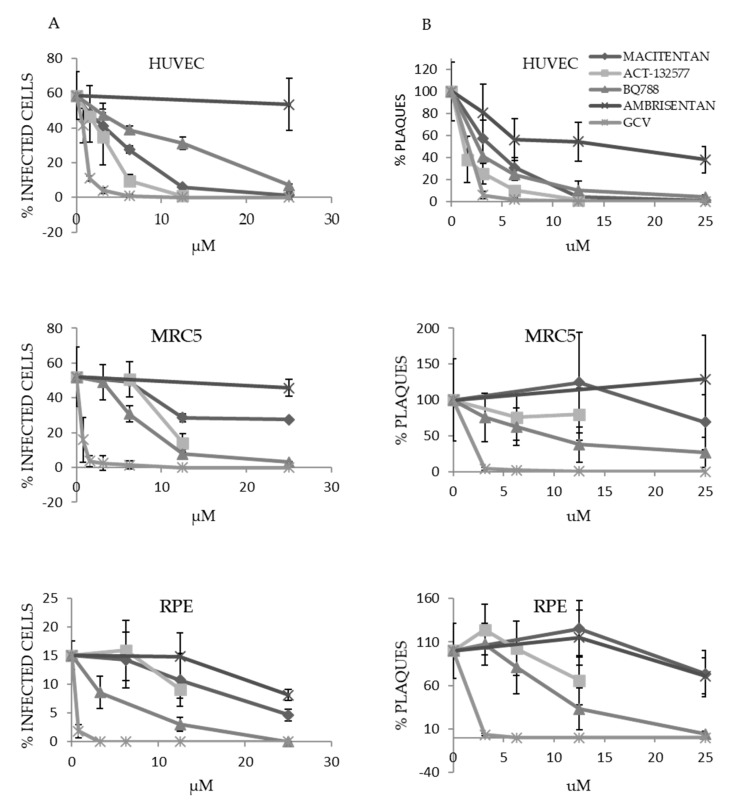
ETR antagonists at low concentrations inhibit production of HCMV in various cell types. HUVECs, MRC5 and RPE-1 cells were treated with low concentrations of ETR antagonists or ganciclovir (GCV) and exposed to HCMV. Seven days later, the virus-containing supernatant was collected or, in the case of RPEs, viral particles were extracted from cells by sonication. The harvested virus containing supernatant was used to infect MRC5 cells. (**A**) The percentage of IE-positive MRC5 cells was determined one day after infection. (**B**) The harvested virus was subjected to serial dilution and titered by a plaque assay in MRC5 cells. Two hours after infection, the supernatant was replaced with methylcellulose-containing medium. At 14 days post infection, the number of plaques formed was quantified from wells where plaques were easy to distinguish, and the titers were calculated. Values are presented as percentage of control without drugs (mean ± SD).

Next, we treated HUVECs, MRC5 and RPE-1 cells with macitentan, ACT-132577, BQ788, ambrisentan or ganciclovir, and thereafter infected them with HCMV. Seven days later, we collected the virus-containing supernatants (HUVECs and MRC5 fibroblasts) or virus-containing cell lysates (RPE-1 cells) and used them to infect MRC5 cells (Figure 3A). We judged viral output from the percentage of IE-expressing cells one day after infection (Figure 4A, Table 3). We also titered the virus-containing supernatants and lysates with a plaque assay (Figure 4B). Ambrisentan did not inhibit virus production in HUVECs or in MRC5 cells and was the least effective inhibitor in RPE-1 cells. BQ788 was the most efficient compound that inhibited virus production in MRC5 and RPE-1 cells and at 25 μM, it essentially blocked virus production in all cell types. Interestingly, we observed that the number of IE positive cells only trended to be reduced (Figure 3E), but the IE staining pattern was completely different in BQ788 treated cells (Figure 3F), which suggest that ETBR signaling is linked to regulation of HCMV IE expression and possibly to control of HCMV replication. At 12.5 μM, macitentan and ACT-132577 reduced virus production by 98–100% in HUVECs, respectively.

### 3.5. Macitentan, Its Metabolite ATC-132577 and BQ788 Inhibit Infection of a Ganciclovir-Resistant HCMV Strain

**Figure 5:**
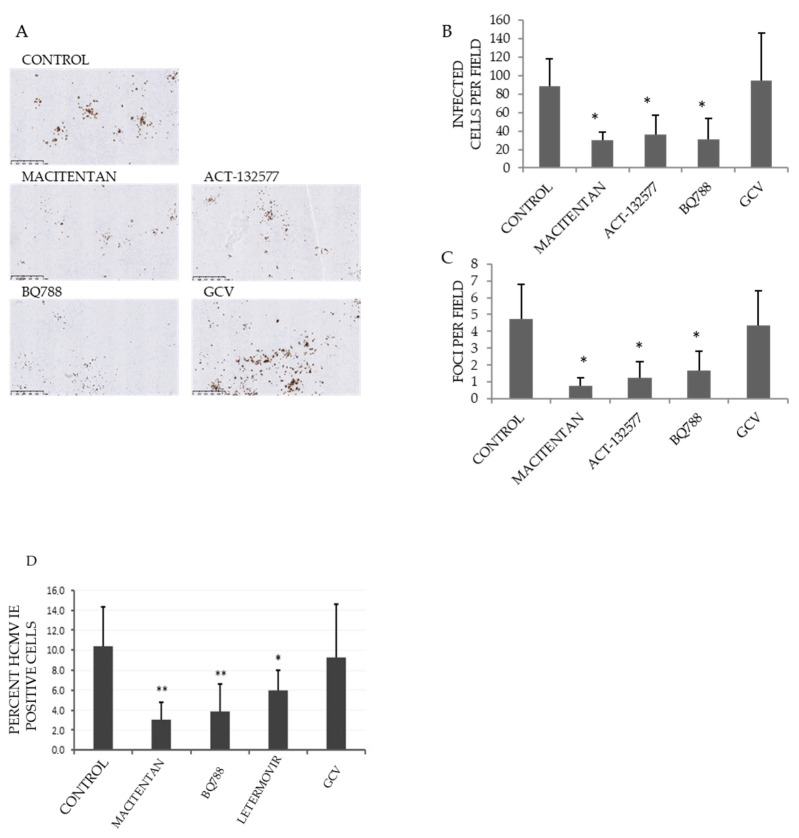
Decreased HCMV-IE positive cells in cultures treated with macitentan, its metabolite ATC-132577 and BQ788. HUVECs were treated with ETR antagonists (12.5 μM) and exposed to MRC5s infected with the ganciclovir-resistant HCMV clinical isolate C17222. Seven days later, IE expression was assessed by immunostaining. (**A**) Representative images. Brown stain corresponds to IE immunoreactivity and represent HCMV positive cells. (**B**) The number of IE-immunopositive cells per field of view was quantified. (**C**) The number of IE-positive foci (comprising more than 3 IE-positive cells) per field of view was quantified. (**D**) HUVEC cells were infected by exposing them to C17222 infected MRC5s (HUVEC: infected MRC5 ratio of 1:1) and treated or not with 10 µM of ganciclovir, 10 µM of letermovir, 100 µM of BQ788 or 25 µM of macitentan. At seven days post infection, the virus-containing cells were harvested and used to infect MRC5 cells. Percentage of positive IE stained cells was measured at 3 dpi. Values are mean ± SD of at least triplicate images. Asterisks (*) denote a statistically significant difference with respect to the control ((*) *p*-value < 0.05 and (**) a *p*-value < 0.01).

To determine whether ETR antagonists could be used as an alternative therapy for ganciclovir-resistant HCMV infections, we treated HUVECs with ETR antagonists and exposed them to MRC5s that were infected with a ganciclovir-resistant HCMV clinical isolate (C17222). This HCMV strain contained the viral UL97 kinase amino acid changes A594V and L595S, which confers ganciclovir resistance [35]. The C17222 virus is highly cell associated, wherefore we used infected MRC-5 cells to infect HUVECs. At the same time, we treated cells with 10 µM of ganciclovir, 10 µM of letermovir, 100 µM of BQ788 or 25 µM of Macitentan, respectively. Three days after infection, we assessed the level of IE expression in HCMV infected cells by immunostaining. We found that macitentan, its metabolite ATC-132577 and BQ788 decreased IE positive cells by 60 to 67% (Figure 5A,B,D) and by 42% with letermovir (5D) compared to controls in cultures infected with the ganciclovir-resistant HCMV isolate. We observed significantly less IE-positive foci in HUVECs treated with ETR antagonists (Figure 5C) or letermovir (Figure 5D), while ganciclovir, as expected, did not affect infection with this ganciclovir-resistant HCMV strain (Table 4).

## 4. Discussion

This study shows that the FDA-approved drug macitentan, its metabolite ACT-132577, and the ETBR-specific antagonist BQ788 are highly effective to inhibit HCMV replication and spread in different human cell types. Macitentan also inhibited infection with a ganciclovir-resistant strain, which is generally very difficult to treat and lacks good treatment options for patients. The effect of macitentan on infection with a ganciclovir-resistant HCMV strain was similar or slightly better than the most recently approved antiviral drug against HCMV; letermovir. Therefore, macitentan may serve as a novel drug with potential efficacy in the treatment of HCMV infections including those infected with a ganciclovir-resistant strain.

The most common cell types infected during acute HCMV disease are macrophages, endothelial cells and epithelial cells. These cell types are relevant during both acute and late phases of infection due to their role in viral dissemination and pathogenesis. The choice of HUVECs, MRC5 and RPE-1 cells also allowed us to compare the outcome of ETR blockade on cells with different modes of viral entry. Specific glycoprotein complexes that are present in the virus envelope bind to particular receptors on target cells, which determine, at least in part, cellular viral tropism [43]. Entry can occur through fusion of the virion with the cellular plasma membrane in a pH-independent manner, as in fibroblasts [44], or through a pH-dependent receptor-mediated endocytosis, which occurs in monocyte/macrophages, endothelial and epithelial cells [9,40,45,46].

HCMV infection is initiated by tethering of the virus to heparin sulphate proteoglycans. The gM/gN heterodimer and the gB protein bind to heparin, whereafter the virus docks to more stable virus receptor interactions before fusion and release of viral components to the cytoplasm takes place. This can occur either at the plasma membrane or after receptor mediated endocytosis, which is followed by intracellular fusion of the viral envelope with the endosome. Several different receptors including platelet-derived growth factor receptor-α (PDGFR-α) [47], integrins (especially subtype αVβ3) [48,49], epidermal growth factor receptor (EGFR) [50], CD13 [51], Neuropilin-2 [52], CD147 [53] and OR14I1 [54] have been shown to mediate entry into different cell types. Two glycoprotein complexes appear to be the main viral components mediating virus cellular tropism. The gH/gL/gO trimer engages PDGFR-α [55] and mediates infection into fibroblasts by initiating fusion at the plasma membrane, but this protein complex does not support HCMV entry into monocytes, endothelial or epithelial cells [56]. In contrast, HCMV enters monocyte/macrophages, endothelial and epithelial cells by an interaction between the gH/gL/UL128/UL130/UL131A pentamer complex and specific receptors on these cell types that mediate receptor mediated endocytosis [52,53,54]. The pentamer complex receptor was difficult to identify, but recent studies suggest that Neuropilin-2 [52], OR14I1 [54] or CD147 [53] can serve as receptors for the pentamer complex.

The pentamer also activates EGFR, and pharmacological inhibition of EGFR signaling hampers translocation of the viral DNA to the nucleus in CD34+ cells. This led to the hypothesis that EGFR activation is involved in pentamer dependent virus entry into myeloid lineage cells [57]. In this context, it is interesting to note that endothelin activation of ETBR transactivates EGFR [58]. Thus, if HCMV interacts with ETBR, this may also result in EGFR activation. Furthermore, when HCMV attaches to target cells, it also elicits a potent cellular interferon-like response, which results in activation of downstream growth factor-like receptor tyrosine kinase (RTK) and integrin pathways [49,59]. Both EGFR and PDGFR, in conjunction with αvβ3 integrins, activate downstream signaling via PI3K/Akt, phospholipase Cγ and focal adhesion kinase [47,48,49,50] and trigger endosome formation and virus uptake. In monocytes, engagement of the viral pentamer complex with an unidentified cellular receptor results in engagement of integrins, src and paxillin, which is followed by activation of an actin and dynamin process that also promotes endocytosis of the viral particle for later intracellular fusion and capsid release [60]. EGFR and downstream PI3K signaling are also important to mediate infection that leads to establishment of latency in CD34+ cells [57]. Thus, an interaction between HCMV and its receptors will result in activation of intracellular signaling pathways that are critical for both latent infection and efficient virus production.

In the present study, we provide evidence that macitentan, its metabolite ATC-132577 and BQ788, all targeting ETBR and prevent HCMV infection. Although it cannot be excluded that binding to ETBR is relevant for HCMV entry, ETBR appears to mediate post-entry cellular activation that is necessary for productive HCMV infection, perhaps by controlling IE expression. With an MOI = 1, we observed very little, if any, inhibition of BQ788 in infected MRC5, which suggests post-entry effects of this drug in this cell type at a low MOI (unpublished data). It is possible that ETBR -mediated cellular signaling triggers transcription of viral or cellular genes that favors completion of the viral life cycle. We provide evidence that the expression pattern of IE is completely different in BQ788 treated infected cells, which suggest that ETBR signaling is connected with regulation of IE expression that could have a profound effect on virus replication. It is also possible that interactions between HCMV proteins and ETBR take place in intracellular compartments or that the interaction between HCMV and ETBR leads to activation of intracellular mechanisms of crucial relevance for HCMV replication. As ETBR inhibitors could also affect HCMV replication after infection, common signaling pathways activated by binding of HCMV to receptors on fibroblasts, endothelial and epithelial cells may be similar to those induced by ETBR ligand interaction. HCMVs ability to activate intracellular signaling pathways is however complex and still poorly understood in the context of promoting HCMV replication.

Today, clinical management of HCMV infections is dependent on effective anti-viral therapies, and multiple options for antiviral therapy of HCMV infections in patients are therefore warranted. An emerging problem of acyclovir- and ganciclovir-resistant strains has fueled the development of new anti-virals. Letermovir, which is the latest approved antiviral drug against HCMV acts on ganciclovir-resistant strains. We found that macitentan compared equal or slightly better than letermovir at tested concentrations to inhibit HCMV infection in vitro, wherefore this drug may provide one additional option for treatment of patients infected with ganciclovir-resistant HCMV strains. Whether or not letermovir or ETR antagonist-resistant HCMV strains could emerge under long term culture or during treatment in patients, is unknown and will require evaluation in future studies.

In addition to an important role of antiviral compounds to combat HCMV infections, immunotherapy strategies have been evaluated for treatment of HCMV disease in transplant patients and for HCMV-positive glioblastoma. HCMV vaccines are also under evaluation for congenitial infections. Development of vaccines have been hampered by the lack of knowledge of the mechanisms of entry into monocyte/macrophages, endothelial cells and epithelial cells, as these cells serve as primary virus targets in early infections. This knowledge has increased in recent years, and our results presented here demonstrate that targeting ETBR is an additional potential new therapeutic option for HCMV infected patients, even for those infected with ganciclovir-resistant strains. Findings from our study suggest that binding or signaling through ETBR is essential for the replication and spread of HCMV. Thus, we provide proof-of-concept evidence that an FDA-approved drug for treatment of PAH may be possible to repurpose for prevention and treatment of HCMV infections. We have observed that inhibition of ETBR fails to inhibit murine or rat CMV infection in mouse or rat cells, respectively, making our study not amenable for testing in common laboratory in vivo models (unpublished results). We have therefore based our conclusions on results obtained from clinically relevant cell types as in vivo preclinical models are unavailable, owing to the selective tropism of HCMV for human cells. Whether our in vitro results mirror any clinical significance remains to be proven in future clinical trials.

## 5. Conclusions

We demonstrate that an FDA approved ETBR inhibitor can prevent HCMV infection in vitro in clinically relevant cell types. This discovery is relevant as novel therapeutic options for HCMV are needed. Ganciclovir-resistant strains are on the rise, and ganciclovir has myelosuppressive effects that are highly undesirable in immunocompromised stem cell transplant recipients. Letermovir, which targets the viral terminase complex [21] was approved for HCMV prophylaxis and treatment in 2017 [22]. We found that macitentan compared equal or slightly better than letermovir to inhibit HCMV infection in vitro with a ganciclovir-resistant strain. Other investigational drugs that target other viral mechanisms have been tested with variable results [15]. Brincidofovir, which also inhibits the HCMV DNA polymerase, failed in a phase III study evaluating its prophylactic effect in stem cell transplant patients [61]. Maribavir failed to show an effect as a prophylactic drug, but is under evaluation for pre-emptive treatment and treatment of established HCMV disease (NCT 02927067 and NCT02931539). Additional preclinical and early clinical trials are therefore warranted to assess the possibility of repurposing macitentan to treat HCMV infections.

## 6. Patents

A patent application for the use of ETBR inhibitors in the treatment of HCMV infections was filed, but was later dropped.

## Figures and Tables

**Table 1:** Treatment of HCMV infected MRC-5, HUVEC and RPE cells with ETR Antagonists or Ganciclovir at different concentrations and cellular viability was quantified.

	MRC5		HUVEC				RPE	
	Concentrations		Concentrations				Concentrations	
Compounds	12.5 µM	25.0 µM	3.2 µM	6.25 µM	12.5 µM	25.0 µM	12.5 µM	25.0 µM
Macitentan	ns	ns	-	ns	ns	ns	****	****
ACT-132577	ns	ns	*	***	****	-	****	****
BQ788	ns	ns	-	ns	ns	ns	****	****
Bosentan	*	****	-	ns	ns	*	ns	****
Ganciclovir	ns	ns	-	ns	ns	ns	ns	ns
Ambrisentan	**	*	-	ns	ns	ns	*	ns

Values are mean ± SD of triplicates. ns; not significant, -; not done. ****; *p* < 0.0001 ***; *p* < 0.001; ** *p* < 0.01, *; *p* < 0.05.

**Table 2:** Pre and post treatment of HCMV infected HUVECs with Ambrisentan, and ETAR- antagonists at different concentrations.

	PRE-TREATMENT			POST-TREATMENT		
Compounds	6.25 µM	12.5 µM	25 µM	6.25 µM	12.5 µM	25.0 µM
Macitentan	*	***	***	*	****	****
ACT-132577	***	***	***	***	****	****
BQ788	ns	ns	**	*	***	***
Bosentan	ns	***	ns	ns	ns	ns
Ambrisentan	***	ns	ns	ns	ns	ns

ns; no significance, -; not done. ****; *p* < 0.0001 ***; *p* < 0.001; ** *p* < 0.01, *; *p* < 0.05.

**Table 3:** HCMV infected HUVECs, MRC5 and RPE were treated with different concentrations of ETR antagonists or Ganciclovir and viral output was calculated from the percentage of IE-expressing cells one day after infection. These results were obtained from data in Figure 4.

Viral output from different cell lines as determined by IE-positive staining in MRC5 cells						
	HUVEC					
Compounds	0.8 µM	1.6 µM	3.0 µM	6.25 µM	12.5 µM	25.0 µM
Macitentan	ns	ns	ns	****	****	****
ACT-132577	ns	ns	****	****	****	****
BQ788	-	ns	ns	*	***	****
Ambrisentan	-	-	-	-	-	ns
Ganciclovir	ns	****	****	****	****	****
	MRC5					
Compounds	0.8 µM	1.6 µM	3.0 µM	6.3 µM	12.5 µM	25.0 µM
Macitentan	-	-	-	ns	**	**
ACT-132577	-	-	-	ns	****	ns
BQ788	-	-	ns	*	****	****
Ambrisentan	-	-	-	-	-	ns
Ganciclovir	****	****	****	****	****	****
	RPE					
Compounds	0.8 µM	3.0 µM	6.3 µM	12.5 µM	25.0 µM	
Macitentan	-	-	ns	ns	***	
ACT-132577	-	-	ns	ns	**	
BQ788	-	ns	-	***	****	
Ambrisentan	-	-	-	ns	*	
Ganciclovir	****	****	-	-	-	
**Viral output from different cells lines as determined by plaque formation in MRC5 cells**						
	HUVEC					
Compounds	1.6 µM	3.0 µM	6.25 µM	12.5 µM	25.0 µM	
Macitentan	-	****	****	****	****	
ACT-132577	****	****	****	****	****	
BQ788	****	****	****	****	****	
Ambrisentan	ns	****	****	****	****	
Ganciclovir	****	****	****	****	****	
	MRC5					
Compounds	1.6 µM	3.0 µM	6.3 µM	12.5 µM	25.0 µM	
Macitentan	-	-	-	ns	ns	
ACT-132577	-	-	-	ns	ns	
BQ788	-	ns	ns	*	**	
Ambrisentan	-	-	-	-	ns	
Ganciclovir	-	****	****	****	****	
	RPE					
Compounds	1.6 µM	3.0 µM	6.3 µM	12.5 µM	25.0 µM	
Macitentan	-	-	-	ns	ns	
ACT-132577	-	ns	ns	ns	-	
BQ788	-	ns	ns	***	****	
Ambrisentan	-	-	-	ns	ns	
Ganciclovir	****	****	****	****	****	

ns; no significance. ****; *p* < 0.0001 ***; *p* < 0.001; ** *p* < 0.01, *; *p* < 0.05.

**Table 4:** EC _50_ for inhibition of infectious viral production.

	HUVEC		RPE-1		MRC5	
**Compound**	EC_50_ (µM)	R^2^	EC_50_ (µM)	R^2^	EC_50_ (µM)	R^2^
**Macitentan**	0.05	0.66	17.30	0.60	20.49	0.33
	4.32	0.81	9.90	0.63	23.95	0.55
**ACT-132577**	1.01	0.66	29.16	0.28	5.66	0.59
	2.75	0.78	9.58	0.50	12.26	0.44
**BQ-788**	3.61	0.66	3.32	0.90	1.66	0.91
	10.07	0.80	9.90	0.58	6.08	0.72
**Ganciclovir**	0.49	0.80	0.09	0.97	0.22	0.88
	0.68	0.87	0.66	0.83	0.24	0.86

Two EC 50 values for each cell-drug combination derived from two independent experiments.

## Data Availability

The datasets generated during the present study are not publicly available, but are available from the corresponding author upon reasonable request.

## References

[1] Boppana S.B., Britt W.J., Reddehase M.J. (2013). Synopsis of Clinical Aspects of Human Cytomegalovirus Diseases.

[2] Ariza-Heredia E.J., Nesher L., Chemaly R.F. (2014). Cytomegalovirus diseases after hematopoietic stem cell transplantation: A mini-review. Cancer Lett..

[3] Eid A.J., Razonable R.R. (2010). New developments in the management of cytomegalovirus infection after solid organ transplantation. Drugs.

[4] Söderberg-Nauclér C., Fish K.N., Nelson J.A. (1997). Reactivation of latent human cytomegalovirus by allogeneic stimulation of blood cells from healthy donors. Cell.

[5] Söderberg-Nauclér C., Fish K.N., Nelson J.A. (1997). Interferon-gamma and tumor necrosis factor-alpha specifically induce formation of cytomegalovirus-permissive monocyte-derived macrophages that are refractory to the antiviral activity of these cytokines. J. Clin. Investig..

[6] Reeves M., Sissons P., Sinclair J. (2005). Reactivation of human cytomegalovirus in dendritic cells. Discov. Med..

[7] Einsele H., Mielke S., Grigoleit G.U. (2014). Diagnosis and treatment of cytomegalovirus 2013. Curr. Opin. Hematol..

[8] Twite N., Andrei G., Kummert C., Donner C., Perez-Morga D., De Vos R., Snoeck R., Marchant A. (2014). Sequestration of human cytomegalovirus by human renal and mammary epithelial cells. Virology.

[9] Sinzger C., Grefte A., Plachter B., Gouw A., The T., Jahn G. (1995). Fibroblasts, epithelial cells, endothelial cells and smooth muscle cells are major targets of human cytomegalovirus infection in lung and gastrointestinal tissues. J. Gen. Virol..

[10] Jabs D.A., Enger C., Dunn J.P., Forman M. (1998). Cytomegalovirus retinitis and viral resistance: Ganciclovir resistance. CMV Retinitis and Viral Resistance Study Group. J. Infect. Dis..

[11] Scholz M., Doerr H.W., Cinatl J. (2003). Human cytomegalovirus retinitis: Pathogenicity, immune evasion and persistence. Trends Microbiol..

[12] Popovic M., Smiljanic K., Dobutovic B., Syrovets T., Simmet T., Isenovic E.R. (2012). Human cytomegalovirus infection and atherothrombosis. J. Thromb Thrombolysis.

[13] Hamilton S.T., van Zuylen W., Shand A., Scott G.M., Naing Z., Hall B., Craig M.E., Rawlinson W.D. (2014). Prevention of congenital cytomegalovirus complications by maternal and neonatal treatments: A systematic review. Rev. Med. Virol..

[14] Johnsen J.I., Baryawno N., Söderberg-Nauclér C. (2011). Is human cytomegalovirus a target in cancer therapy?. Oncotarget.

[15] Mercorelli B., Lembo D., Palu G., Loregian A. (2011). Early inhibitors of human cytomegalovirus: State-of-art and therapeutic perspectives. Pharmacol. Ther..

[16] Gilbert C., Boivin G. (2005). Human cytomegalovirus resistance to antiviral drugs. Antimicrob. Agents Chemother..

[17] Foulongne V., Turriere C., Diafouka F., Abraham B., Lastere S., Segondy M. (2004). Ganciclovir resistance mutations in UL97 and UL54 genes of Human cytomegalovirus isolates resistant to ganciclovir. Acta Virol..

[18] Smith I.L., Cherrington J.M., Jiles R.E., Fuller M.D., Freeman W.R., Spector S.A. (1997). High-level resistance of cytomegalovirus to ganciclovir is associated with alterations in both the UL97 and DNA polymerase genes. J. Infect. Dis..

[19] Torres-Madriz G., Boucher H.W. (2008). Immunocompromised hosts: Perspectives in the treatment and prophylaxis of cytomegalovirus disease in solid-organ transplant recipients. Clin. Infect. Dis..

[20] Erice A. (1999). Resistance of human cytomegalovirus to antiviral drugs. Clin. Microbiol. Rev..

[21] Melendez D.P., Razonable R.R. (2015). Letermovir and inhibitors of the terminase complex: A promising new class of investigational antiviral drugs against human cytomegalovirus. Infect. Drug Resist..

[22] Imlay H.N., Kaul D.R. (2020). Letermovir and Maribavir for the Treatment and Prevention of Cytomegalovirus Infection in Solid Organ and Stem Cell Transplant Recipients. Clin. Infect. Dis..

[23] Yaiw K.-C., Mohammad A.-A., Costa H., Taher C., Badrnya S., Assinger A., Wilhelmi V., Ananthaseshan S., Estekizadeh A., Davoudi B. (2015). Human Cytomegalovirus Up-Regulates Endothelin Receptor Type B: Implication for Vasculopathies?. Open Forum. Infect. Dis..

[24] Yaiw K.C., Mohammad A.A., Taher C., Cui H.L., Costa H., Kostopoulou O.N., Jung M., Assinger A., Wilhelmi V., Yang J. (2021). Human Cytomegalovirus Reduces Endothelin-1 Expression in Both Endothelial and Vascular Smooth Muscle Cells. Microorganisms.

[25] Dupuis J., Hoeper M.M. (2008). Endothelin receptor antagonists in pulmonary arterial hypertension. Eur. Respir. J..

[26] Luscher T.F., Barton M. (2000). Endothelins and endothelin receptor antagonists: Therapeutic considerations for a novel class of cardiovascular drugs. Circulation.

[27] Hoeper M.M., McLaughlin V.V., Dalaan A.M., Satoh T., Galie N. (2016). Treatment of pulmonary hypertension. Lancet Respir Med..

[28] Enderby C.Y., Burger C. (2015). Medical treatment update on pulmonary arterial hypertension. Ther. Adv. Chronic. Dis..

[29] Zebadua R., Hernandez-Perez A.P., Garcia A., Zayas N., Sandoval J., Lopez J., Pulido T. (2021). Macitentan in the treatment of pulmonary arterial hypertension. Future Cardiol..

[30] Savale L., Magnier R., Le Pavec J., Jais X., Montani D., O’Callaghan D.S., Humbert M., Dingemanse J., Simonneau G., Sitbon O. (2013). Efficacy, safety and pharmacokinetics of bosentan in portopulmonary hypertension. Eur. Respir. J..

[31] Kingman M., Ruggiero R., Torres F. (2009). Ambrisentan, an endothelin receptor type A-selective endothelin receptor antagonist, for the treatment of pulmonary arterial hypertension. Expert Opin. Pharmacother..

[32] Murrell I., Bedford C., Ladell K., Miners K.L., Price D.A., Tomasec P., Wilkinson G.W.G., Stanton R.J. (2017). The pentameric complex drives immunologically covert cell-cell transmission of wild-type human cytomegalovirus. Proc. Natl. Acad. Sci. USA.

[33] Miceli M.V., Newsome D.A., Novak L.C., Beuerman R.W. (1989). Cytomegalovirus replication in cultured human retinal pigment epithelial cells. Curr. Eye Res..

[34] Cooke B.M., Usami S., Perry I., Nash G.B. (1993). A simplified method for culture of endothelial cells and analysis of adhesion of blood cells under conditions of flow. Microvasc. Res..

[35] Rosen H.R., Benner K.G., Flora K.D., Rabkin J.M., Orloff S.L., Olyaei A., Chou S. (1997). Development of ganciclovir resistance during treatment of primary cytomegalovirus infection after liver transplantation. Transplantation.

[36] Awasthi S., Isler J.A., Alwine J.C. (2004). Analysis of splice variants of the immediate-early 1 region of human cytomegalovirus. J. Virol..

[37] Reed L.J., Muench H. (1938). A simple method of estimating fifty per cent endpoints. Am. J. Epidemiol..

[38] Sidharta P.N., Treiber A., Dingemanse J. (2015). Clinical pharmacokinetics and pharmacodynamics of the endothelin receptor antagonist macitentan. Clin. Pharmacokinet.

[39] Weber C., Gasser R., Hopfgartner G. (1999). Absorption, excretion, and metabolism of the endothelin receptor antagonist bosentan in healthy male subjects. Drug Metab. Dispos..

[40] Bodaghi B., Slobbe-van Drunen M.E., Topilko A., Perret E., Vossen R.C., van Dam-Mieras M.C., Zipeto D., Virelizier J.L., LeHoang P., Bruggeman C.A. (1999). Entry of human cytomegalovirus into retinal pigment epithelial and endothelial cells by endocytosis. Investig. Ophthalmol. Vis. Sci..

[41] Topilko A., Michelson S. (1994). Hyperimmediate entry of human cytomegalovirus virions and dense bodies into human fibroblasts. Res. Virol..

[42] Detrick B., Rhame J., Wang Y., Nagineni C.N., Hooks J.J. (1996). Cytomegalovirus replication in human retinal pigment epithelial cells. Altered expression of viral early proteins. Investig. Ophthalmol. Vis. Sci..

[43] Vanarsdall A.L., Johnson D.C. (2012). Human cytomegalovirus entry into cells. Curr. Opin. Virol..

[44] Compton T., Nepomuceno R.R., Nowlin D.M. (1992). Human cytomegalovirus penetrates host cells by pH-independent fusion at the cell surface. Virology.

[45] Dankner W.M., McCutchan J.A., Richman D.D., Hirata K., Spector S.A. (1990). Localization of human cytomegalovirus in peripheral blood leukocytes by in situ hybridization. J. Infect. Dis..

[46] Jarvis M.A., Nelson J.A. (2007). Human cytomegalovirus tropism for endothelial cells: Not all endothelial cells are created equal. J. Virol..

[47] Soroceanu L., Akhavan A., Cobbs C.S. (2008). Platelet-derived growth factor-alpha receptor activation is required for human cytomegalovirus infection. Nature.

[48] Wang X., Huang D.Y., Huong S.M., Huang E.S. (2005). Integrin alphavbeta3 is a coreceptor for human cytomegalovirus. Nat. Med..

[49] Feire A.L., Koss H., Compton T. (2004). Cellular integrins function as entry receptors for human cytomegalovirus via a highly conserved disintegrin-like domain. Proc. Natl. Acad. Sci. USA.

[50] Wang X., Huong S.M., Chiu M.L., Raab-Traub N., Huang E.S. (2003). Epidermal growth factor receptor is a cellular receptor for human cytomegalovirus. Nature.

[51] Söderberg C., Giugni T.D., Zaia J.A., Larsson S., Wahlberg J.M., Moller E. (1993). CD13 (human aminopeptidase N) mediates human cytomegalovirus infection. J. Virol..

[52] Martinez-Martin N., Marcandalli J., Huang C.S., Arthur C.P., Perotti M., Foglierini M., Ho H., Dosey A.M., Shriver S., Payandeh J. (2018). An Unbiased Screen for Human Cytomegalovirus Identifies Neuropilin-2 as a Central Viral Receptor. Cell.

[53] Vanarsdall A.L., Pritchard S.R., Wisner T.W., Liu J., Jardetzky T.S., Johnson D.C. (2018). CD147 Promotes Entry of Pentamer-Expressing Human Cytomegalovirus into Epithelial and Endothelial Cells. mBio.

[54] E X., Meraner P., Lu P., Perreira J.M., Aker A.M., McDougall W.M., Zhuge R., Chan G.C., Gerstein R.M., Caposio P. (2019). OR14I1 is a receptor for the human cytomegalovirus pentameric complex and defines viral epithelial cell tropism. Proc. Natl. Acad. Sci. USA.

[55] Kabanova A., Marcandalli J., Zhou T., Bianchi S., Baxa U., Tsybovsky Y., Lilleri D., Silacci-Fregni C., Foglierini M., Fernandez-Rodriguez B.M. (2016). Platelet-derived growth factor-alpha receptor is the cellular receptor for human cytomegalovirus gHgLgO trimer. Nat. Microbiol..

[56] Vanarsdall A.L., Howard P.W., Wisner T.W., Johnson D.C. (2016). Human Cytomegalovirus gH/gL Forms a Stable Complex with the Fusion Protein gB in Virions. PLoS Pathog..

[57] Kim J.H., Collins-McMillen D., Buehler J.C., Goodrum F.D., Yurochko A.D. (2017). Human Cytomegalovirus Requires Epidermal Growth Factor Receptor Signaling To Enter and Initiate the Early Steps in the Establishment of Latency in CD34+ Human Progenitor Cells. J. Virol..

[58] Moody T.W., Ramos-Alvarez I., Moreno P., Mantey S.A., Ridnour L., Wink D., Jensen R.T. (2017). Endothelin causes transactivation of the EGFR and HER2 in non-small cell lung cancer cells. Peptides.

[59] Compton T. (2004). Receptors and immune sensors: The complex entry path of human cytomegalovirus. Trends Cell Biol..

[60] Nogalski M.T., Chan G.C., Stevenson E.V., Collins-McMillen D.K., Yurochko A.D. (2013). The HCMV gH/gL/UL128-131 complex triggers the specific cellular activation required for efficient viral internalization into target monocytes. PLoS Pathog.

[61] Alvarez-Cardona J.J., Whited L.K., Chemaly R.F. (2020). Brincidofovir: Understanding its unique profile and potential role against adenovirus and other viral infections. Future Microbiol..

